# How Are Diagnosis-Related Groups and Staffing Allocation Systems Associated with the Complexity of Nursing Care? An Observational Study

**DOI:** 10.3390/healthcare12191988

**Published:** 2024-10-05

**Authors:** Diletta Fabrizi, Davide Paolo Bernasconi, Giulia Locatelli, Michela Luciani, Giorgio Beretta, Stefania Di Mauro, Paola Rebora, Davide Ausili

**Affiliations:** 1 Department of Medicine and Surgery, University of Milano-Bicocca, Via Cadore, 48, 20900 Monza, Italy; diletta.fabrizi@unimib.it (D.F.); davide.bernasconi@unimib.it (D.P.B.); michela.luciani@unimib.it (M.L.); stefania.dimauro@unimib.it (S.D.M.); paola.rebora@unimib.it (P.R.); davide.ausili@unimib.it (D.A.); 2 Bicocca Bioinformatics Biostatistics and Bioimaging (B4) Centre, School of Medicine and Surgery, University of Milano-Bicocca, Via Follereau 3, 20854 Vedano al Lambro, Italy; 3 Clinical Research and Innovation Unit, ASST Grande Ospedale Metropolitano Niguarda, Piazza dell’Ospedale Maggiore, 3, 20162 Milano, Italy; 4 Direzione Aziendale delle Professioni Sanitarie e Socio-Sanitarie, Azienda Socio Sanitaria Territoriale di Lecco, Via dell’Eremo 9/11, 23900 Lecco, Italy; g.beretta@asst-lecco.it; 5 Biostatistics and Clinical Epidemiology, Fondazione IRCCS San Gerardo dei Tintori, Via Pergolesi 33, 20900 Monza, Italy

**Keywords:** complexity of nursing care, diagnosis-related groups, nursing staff, financial system, National Health Service, nursing care, financial management

## Abstract

Background: In Italy, Diagnosis-Related Groups (DRGs) have been adopted for hospital services reimbursement. In some Italian regions, nurse staffing allocation is purely volume-based, with different minutes/patient/day categories determined by the type of hospital ward. The Information System of Nursing Performance (SIPI) is a valid and reliable tool assessing nursing care complexity as an indicator of the actual nursing care demand. Evidence is lacking about the ability of current resource allocation methods to account for the nursing care demand. Objective: To evaluate the association between (1) DRG rates and nursing care complexity and (2) hospital ward categories of nurse staffing and nursing care complexity. Methods: All patients discharged from the medical department of an Italian hospital over a data collection period were eligible. To assess the association between nursing care complexity (SIPI) and DRGs, the distribution of the DRG rate (median and first–third quartile) was compared for cases with high or low complexity. To evaluate the association between nursing care complexity (SIPI) and nurse staffing, the frequency of high complexity within nurse staffing categories (120/180/240 min/patient/day) was compared. Because the sample was very large, methods of statistical inference were not applied, and only descriptive measures were reported. Results: 6872 hospitalizations were included. The median DRG rate for high and low complexity admissions were very similar (EUR 3536 and EUR 3285, respectively). The proportion of admissions with high complexity decreased for wards with higher staffing allocation rates. Conclusion: DRG reimbursement and the nurse staffing allocation systems were ineffective in accounting for nursing care complexity. The SIPI could help identify areas requiring more financial and staffing resources for nursing care.

## 1. Introduction

The Diagnosis-Related Groups (DRG)-based hospital payment system was introduced in the United States in 1983 as a reimbursement model for medical hospital care [1]. DRGs classify patients according to homogeneous economic (e.g., length of stay, charges, etc.) and clinical features (e.g., medical diagnosis, comorbidities, complications, procedures, etc.) [2]. Under this financing system, each DRG corresponds to an established tariff [3]. The DRG system has progressively become the most widespread reimbursement method for acute hospital care [1]. In Italy, the National Health Service (NHS) was established in 1978 with a fee-for-service financing system. The 1992 NHS reform introduced the economic constraint of balancing costs and revenues for the National Health Service, valuing the services provided based on predefined rates. In this context, the system of classifying hospital admissions using DRGs, to which those rates corresponded, was instituted [4]. The classification of hospital admissions is built on the information provided by the Hospital Discharge Form [5]. Specifically, the reimbursement is calculated by associating each hospitalization to a specific DRG category based on the diagnosis and procedures recorded on the Hospital Discharge Form [4]. Each DRG has a predefined tariff that is supposed to reflect the average cost of treating patients within that group [6]. In Italy, the DRG system encompasses 538 final categories of hospitalization, defined as clinically significant and internally homogeneous for resource consumption [6]. However, several studies worldwide showed that DRGs are not able to recognize and account for the variability in nursing care that may occur under the same clinical diagnoses [2,7]. Indeed, the vast majority of hospital diagnoses, therapies, and procedures are related to a specific medical condition, which can often fail to explain the nursing component of care [8]. This could be especially true for aspects of nursing care that are not directly attributable to disease and treatment groups [8]. Consequently, the use of flat per diem charges for nursing care costs has been considered one of the most impactful causes of the mismatch between payments and real costs for inpatients [9].

Besides the financing system, resource allocation in a hospital setting involves the assignment of personnel, including nursing staff. It is known that nurse staffing is associated with relevant outcomes such as mortality, re-hospitalization, adverse events, and length of hospitalization [10,11,12]. Moreover, nurse staffing is strongly related to patients’ satisfaction and missed nursing care [10,12,13,14,15]. These elements suggest that nursing care has a key role in both the safety and quality of hospital care [16]. There are several models for establishing minimum nurse staffing based on the estimated demand for nursing care. These models include (1) professional judgment methods, recently adopted by the United States Veterans’ Administration, which rely on expert opinion without requiring any objective measures to define nursing staffing levels [17]; (2) volume-based approaches, used for instance in California (United States) and in Victoria (Australia), which typically use patient count as the criterion to determine the nursing workload, establishing a minimum nurse–patient ratio [17]; (3) patient/prototype classification systems, such as the Safer Nursing Care Tool adopted in England [18], which classify patients according to their nursing care needs, with a specific staffing level corresponding to each classification [17]; and (4) timed-task methods, such as the General Responsibility Assignment Software Patterns (GRASP) system in the United States, which identify the required tasks for each patient’s care plane, and since each task corresponds to a specific amount of time, they are used to determine the necessary staffing levels [17]. The nurse staffing allocation system in Lombardy (i.e., the Italian region in which this study was carried out) falls under the volume-based approach, setting a target number of nurses or hours per patient to be applied to broad categories in hospital wards [17]. Specifically, the allocation system used in Lombardy cites the intensity of care as its reference criterion and establishes a minimum of minutes per patient per day that ranges from 120 in general wards to 600 in the intensive care area [19]. However, this kind of method based on patient count does not consider that patients both in the same category of hospital wards and in the same unit may have very heterogeneous nursing care requirements [17]. Supplementing purely volume-based allocation systems with the consideration of this variability could provide a more accurate and comprehensive picture of the actual need for nursing care [17]. Methods for classifying patients based on the complexity of nursing care they require could be useful for this aim [20].

The complexity of nursing care is defined as “the whole set of acts that reflect all dimensions of care expressed in terms of intensity, engagement, and quantity of nurses’ work” [20] and serves as a marker of the demand for nursing care based on a patient’s actual needs. The Information System of Nursing Performance (SIPI—in Italian “Sistema Informativo della Performance Infermieristica”) is a survey tool developed in Italy that evaluates whether the patient requires assistance in the key areas that encompass inpatient nursing care, such as breathing, feeding, hydration, urination, defecation, hygiene, mobility, etc. [20]. The SIPI proved to be able to assess and quantify the complexity of nursing care in all specialties, excluding psychiatric and pediatric wards, intensive care units, and outpatient services [20]. Furthermore, the SIPI demonstrated that its score is strongly associated with in-hospital mortality [16], supporting the strong link between nursing care and relevant hospital outcomes [8].

In such a context, the DRG system constitutes the main criterion for allocating financial resources on the one hand, and, on the other hand, minutes per patient per day categories determined by the type of hospital ward establish nurse staffing levels. However, to the best of our knowledge, neither the associations between DRG rates and the complexity of nursing care nor the association between nurse staffing categories and the complexity of nursing care have ever been assessed in the Italian NHS. Thus, we do not have any scientific evidence about the ability or inability of the current resource allocation system to account for real patients’ needs for nursing care. Having this information could be relevant for several reasons. First, it could allow for the development of strategies for the integration or modification of the DRG financing system to better account for nursing care needs. Second, it could inform managers and policy makers about the consistency of the volume-based system used to allocate nurses with the real complexity of nursing care. Third, it could allow for the development of new and flexible ways to determine nurse staffing allocation, considering the complexity of nursing care and not only the ward where patients are admitted. Finally, it could contribute to the international debate, comparisons, and policies on nurse staffing levels and resource utilization in acute care hospitals.

Based on these gaps, the aims of this study were (1) to assess the association between the DRG-based payment system and the complexity of nursing care, as measured by the SIPI score; (2) to assess the association between nurse staffing and the complexity of nursing care (SIPI); and (3) to assess the association between the complexity of nursing care (SIPI) and mortality, accounting for the nurse staffing level and the DRG-based payment system.

## 2. Materials and Methods

This is a secondary analysis of a previous register-based retrospective cohort study aimed at assessing the association between the complexity of nursing care and in-hospital mortality [16]. No further data were collected for this secondary analysis. Authorization was obtained by the Institutional Review Board of the hospital. Informed consent for the use of sociodemographic, clinic, and administrative data for epidemiological purposes was signed by each patient upon admission.

### 2.1. Sample and Setting

The parent study [16] was conducted in a medium-large hospital in Northern Italy, where standard clinical patient documentation included the SIPI. All in-hospital patients discharged from the Medical Center during the 24 months of the data collection period (2014–2015) were eligible for this study. A lack of informed consent and inappropriate admission to the Medical Center were the exclusion criteria. The Medical Center comprised eight clinical units, both generalist and specialist. Specifically, they were three units of the General Medicine ward, collectively comprising 48 beds and having a mean nurse-to-patient ratio of 1:12; one unit of the Admission and Clinical-Care Planning ward (SOAP—in Italian, “Struttura Operativa per Accettazione e Pianificazione clinico-assistenziale”), comprising 30 beds and having a mean nurse-to-patient ratio of 1:10; two units of the Oncology ward, collectively comprising 16 beds and having a mean nurse-to-patient ratio of 1:10; one unit of the Nephrology ward, comprising 25 beds and having a mean nurse-to-patient ratio of 1:10; and one unit of the Infectious Diseases ward, comprising 15 beds and having a mean nurse-to-patient ratio of 1:8.

### 2.2. Variables and Measurements

Sociodemographic and clinical data on age, biological sex, intensity of care in the admission unit (i.e., low or medium), type of admission (i.e., urgent or planned), comorbidities, length of stay, in-hospital death, and DRG rate were collected from the Hospital Discharge Register.

The information about minutes of nursing care needed per patient per day in each of the included wards was provided by the Hospital Nursing Management and was consistent with the reference regional legislation [19]. In the General Medicine ward, the minimum nurse staffing was determined as 120 min per patient per day; in the SOAP, Nephrology, and Oncology wards, it was 180 min per patient per day, and in the Infectious Diseases ward, it was 240 min per patient per day.

The presence of comorbid conditions was assessed through the Charlson Comorbidity Index (CCI). The CCI considers 19 possible comorbidities, with higher scores meaning more comorbidity burden [21]. The complexity of nursing care at admission was measured by using the SIPI, which includes 18 items with a binary response about the presence or absence of each described condition [20]. Specifically, the nursing activities assessed by the SIPI cover the following areas: to ensure breathing (two items), to ensure feeding and hydration (two items), to ensure urination and defecation (two items), to ensure hygiene (two items), to ensure mobility (three items), to monitor cardiac function (one item), to conduct diagnostic procedures (two items), and to apply therapeutic procedures (four items). Each item has its own score assigned only if the answer is positive, i.e., if that activity has been performed and recorded in the nursing file in the last 24 h. The SIPI total score ranges from 0 to 100, with higher scores indicating higher complexity of nursing care. A cut-off value of 50 was chosen to discriminate between patients at a high level of complexity (≥) and patients at a low level of complexity (<) [20]. The complexity of nursing care was documented by nurses in the SIPI database. The SIPI score is recorded at regular time intervals for each admitted patient. For this study, we used the first measurement, registered within the first 24 h from patient admission [16].

### 2.3. Statistical Analysis

Patients’ sociodemographic and clinical data were reported overall, by the complexity of nursing care (i.e., high or low), as measured by the SIPI scores, and by nurse staffing categories (i.e., 120, 180, or 240 min), expressed in minutes per patient. We used the median and interquartile range (IQR) for continuous (non-Gaussian) variables and absolute and percentage frequencies for categorical variables.

To describe the association between the SIPI and the DRG-based payment system (aim 1), we used a boxplot of the DRG rates by the SIPI scores split in deciles. We also compared the median and IQR of DRG rates for SIPI ≥ 50 and <50. To describe the association between SIPI and nurse staffing (aim 2), we used a boxplot of the SIPI score distribution by nurse staffing categories. We also considered the frequency of high complexity (SIPI ≥ 50) in the same categories. We left out the *p*-values of the tests of statistical significance of the differences between groups since, due to the large sample, even small differences without any relevance were statistically significant. The estimates calculated on such a large sample have negligible standard errors and thus could be interpreted as population quantities. Moreover, to assess the relationship between the SIPI, DRG rates, and nurse staffing categories concurrently, we employed a graphical analysis. Specifically, we compared three panels corresponding to the three nurse staffing categories, where the scatter plots with smoothed tendency lines illustrated the association between the SIPI scores (x axis) and DRG rates (y axis).

Furthermore, to assess the association between the SIPI scores and in-hospital mortality holding nurse staffing categories and the DGR-based rate equal (aim 3), we adopted uni- and multivariable Cox models adjusting for the following covariates: age, gender, type of ward (i.e., low intensity vs. medium intensity), type of access (i.e., urgent vs. ordinary), and comorbidities. The Cox model is a semi-parametric regression model that is widely adopted to assess the association of multiple covariates on a time-to-event outcome. It does not require any distributional assumption about the time variable but, for each covariate, assumes a constant effect over time (proportional hazard assumption). We investigated the validity of this assumption for SIPI using Schoenfeld residuals, and it was not tenable. To overcome this issue, the hazard ratio (HR) of SIPI (≥50 vs. <50) was estimated separately within two-time intervals: until and after the 10th day since admission. Further complexity is represented by the presence of multiple admissions for each patient. Thus, to account for the possible within-subject correlations, we included a gamma frailty term in the model [22]. All of the variables considered in this analysis were fully observed, with no missing data.

## 3. Results

The parent study presented a flowchart of hospital admission records and the number of patients analyzed in the study [16]. Briefly, we considered 6872 records referring to 5129 patients, as 1092 patients had multiple hospitalizations.

Overall, the patients had a median age of 76 years (IQR = 64–84) and were mainly males (52%, n = 2657). The admissions were mostly in low-intensity wards (66%), following emergency access (92%), with a median length of 11 days and in-hospital mortality occurring in 7% of cases. The CCI was mostly ≥1 (76%), and the SIPI scores denoted high complexity of nursing care (≥50 point) in 57% of cases. Nurse staffing mostly coincided with 180 min per patient per day (64%), and the median of the DRG rate was EUR 3325. Compared with admissions classified as “low nursing complexity” by the SIPI score, those classified as “high nursing complexity” included older patients (81 vs. 71 years) with more comorbidities (CCI ≥ 2: 38% vs. 27%) and a lower percentage of females (48% vs. 57%) and urgent hospital accesses (97% vs. 87%). Moreover, the corresponding hospital stay was significantly longer (13 vs. 9 days) than the other admissions. Patients’ characteristics at admission, both overall and categorized by the complexity of nursing care, are presented in Table 1.

When comparing patients’ characteristics by the three categories of nurse staffing, the median age of patients decreased with the increase in minutes per patient per day (81 years for the 120′ category, 75 years for the 180′ category, and 57 years for the 240′ category), while the percentage of females increased (49, 53, and 62%). The lowest percentage of emergency admissions corresponded to the 180 min per patient per day category (88% vs. 97% in the 120′ category and 98% in the 240′ category), as well as the lowest length of hospitalization (9 days vs. 14 in the 120′ category and 10 in the 240′ category) and the lowest DRG rate (EUR 3285 vs. EUR 3298 in the 120′ category and 3536 in the 240′ category). The highest percentage of admissions characterized by a CCI of 0 was in the 240 min per patient per day category (50% vs. 23% in the 120′ category and 20% in the 180′ category), while the highest percentage with in-hospital mortality was in the 120 min per patient per day category (8% vs. 7% in the 180′ category and 4% in the 240′ category). Patients’ characteristics by nurse staffing categories are presented in Table 2.

**Figure 1:**
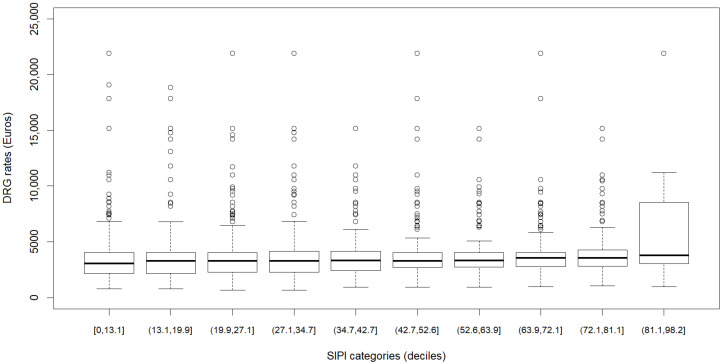
Boxplot showing the distribution of DRG rates in EUR by SIPI categories according to deciles.

The median of the DRG rate for admissions with a SIPI score < 50 was EUR 3285 (IQR = 2298–4052), while for admissions with a SIPI score ≥ 50, it was EUR 3536 (IQR = 2805–4278). More in detail (Figure 1), we found no association between nursing care complexity and DRG rates; the distribution of the DGR rates overlapped for every SIPI category, except for the last decile, where slightly higher DRG rate values were observed. Indeed, the difference in DRG rates between the first and last deciles of the SIPI scores was EUR 727 (Table 3). Consistently, the Spearman index denoted a very weak correlation between the SIPI scores and DRG rates (rho = 0.15, 95% confidence interval (CI): 0.13; 0.17) (Figure S1).

**Figure 2:**
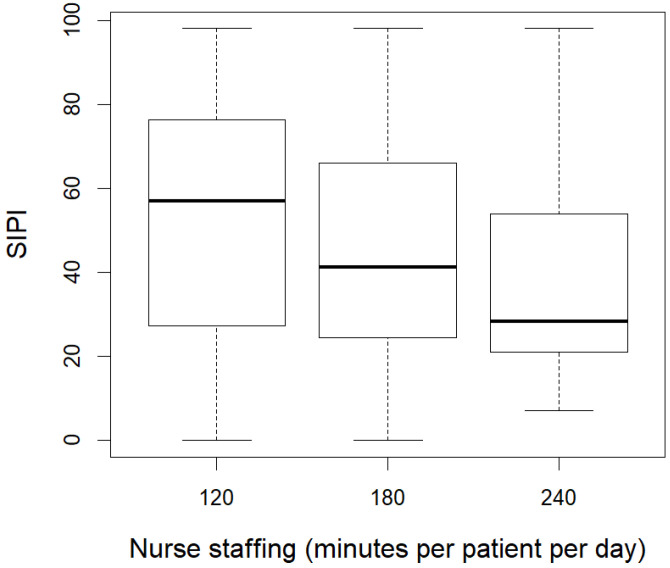
Boxplot showing the distribution of SIPI by nurse staffing (minutes per patient per day).

As shown in Figure 2, higher levels of nursing care complexity corresponded to decreasing categories of nurse staffing. For wards categorized as needing 120 min of nursing care per patient per day, the median of the SIPI score was 57.0 (IQR = 27.4–76.5); for wards with 180 min of nursing care per patient per day, it was 41.3 (IQR = 24.6–66.1); and for the ward with 240 min of nursing care per patient per day, it was 28.3 (IQR = 21.0–53.9). Consistently, the proportion of admissions with a SIPI score ≥ 50 decreased as categories of nursing staffing increased, being 56% for 120, 40% for 180, and 28% for 240 min of nursing care per patient per day.

**Figure 3:**
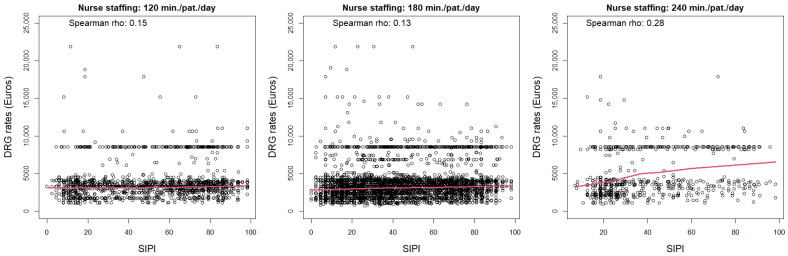
Scatter plots of the association between SIPI and DRG rates across nurse staffing categories (minutes per patient per day). Smoothed tendency lines are also shown in red.

When the relationship between the SIPI, DRG rates, and nurse staffing categories was concurrently assessed, the correlation between SIPI and DRG rates was shown to be very weak across the three nurse staffing categories (Figure 3).

As presented in the parent study [16], over the 6872 admissions, there were 469 in-hospital deaths (7%)—395/2922 (14%) among those with a SIPI score ≥ 50 and 74/3950 (2%) among those with a SIPI score < 50, leading to a difference of 12% (95% CI: 10;13%). As shown in Table 4, the SIPI score at admission was associated with in-hospital mortality (*p* < 0.001), also when adjusting for nurse staffing and DRG rates. Until the 10th day since admission, the estimated hazard rate of mortality in patients with a SIPI score ≥ 50 was 6 times higher than in patients with SIPI score < 50 (HR = 6.22, 95% CI: 4.25; 9.10). After the 10th day since admission, the HR was lower but still higher than one (HR = 2.46, 95% CI: 1.74; 3.46). Considering nurse staffing, the estimated hazard rate of mortality in the 180 min per patient per day wards was 1.5 times higher than in the 120 min per patient per day wards (HR = 1.55, 95% CI: 1.20; 1.99), while the HR between the 240 and 120 min per patient per day wards was not significantly higher than one (HR = 1.09, 95% CI: 0.73; 1.64).

## 4. Discussion

This study aimed to evaluate the association between the complexity of nursing care and both the DRG-based payment system and nurse staffing. We found that the DRG rates nearly overlapped across all levels of complexity of nursing care. Moreover, the increasing complexity of nursing care corresponded to a decrease in nurse staffing. These results could offer the opportunity to reassess the adequacy of the current health financing system and the criteria used for nurse staffing allocation in response to the needs of both the National Health Service and patients. Our findings suggest that incorporating the complexity of nursing care into the definition of these parameters could serve as a pivotal criterion for designing effective resource allocation systems.

We found substantially similar values of DRG rates for increasing levels of nursing complexity. The lack of association between care complexity and the DRG rate confirms the results of previous studies showing the independence of nursing care needs from medical diagnoses [8,23]. This result suggests that nursing care should not be considered just a routine cost in the DRG system, remaining an unquantified value [7,24,25]. Since the complexity of nursing care reflects the nursing workload [20] and predicts relevant patient hospital outcomes [16,23], it should be systematically measured [8] and included among the criteria for functionally allocating financial resources [11].

We also found that the complexity of nursing care increased considerably where the availability of nurse staffing was lower. Particularly, in wards with the lowest nurse staffing (i.e., 120 min per patient per day), the median value of the SIPI score was significantly higher than the cut-point of 50, indicating the high complexity of nursing care [20]. This result suggests that the criterion of “minutes per patient per day” only based on the type of ward does not reflect the actual workload. This allocation method could therefore lead to obstacles in ensuring standards of care tailored to patients’ needs [26,27]. Indeed, previous studies showed that a lack of nurse staffing leads to inadequate nursing care and is associated with preventable adverse patient events, such as a higher occurrence of infections and falls, increased length of stay, and increased in-hospital deaths [12,13,28,29].

On the other hand, the SIPI scores appeared to effectively capture highly relevant features (e.g., patient comorbidities and advanced age, type of admission, and length of stay) that denote a greater need for both financial and staffing resources. Coherently, our data showed that, even with constant values for both DRG rates and nurse staffing, the hazard rate of mortality was higher in patients with high nursing care complexity compared to those with low complexity, confirming the SIPI as a strong independent predictor of mortality [8]. These findings highlight the importance of accounting for nursing care demands by integrating tools like the SIPI into practice to improve decision making, address adverse patient outcomes, and ensure the quality of hospital care [30].

## 5. Conclusions

The DRG-based payment system aims to facilitate hospital reimbursement uniformity by classifying patients according to their diagnosis and comparable clinical characteristics. Furthermore, DRGs assume homogeneity in the demand for nursing care under the same DRG. However, our findings demonstrate that DRG rates are unable to reflect the actual demand for nursing care, as they do not grant the allocation of greater resources to cases requiring higher nursing complexity. Furthermore, this study shows that the current nurse staffing allocation model, based solely on minutes per patient per day by ward type, does not consider the actual nursing care demand attributable to the complexity of nursing care. These results suggest that, in their current form, both the DRG-based system and staffing allocation system might be inadequate, as they fail to distribute resources proportionate to the actual need for such a major component of hospital care as nursing. This misalignment may lead to inadequate patient care and inappropriate workload distribution to nurses and, as a consequence, to avoidable adverse events and poor quality of the provided hospital care. The use of systematic assessments of nursing care complexity, such as the SIPI, could help provide a solid foundation for rethinking and integrating the criteria for financial and staffing resource allocation [16,20].

To support these recommendations, future research should involve broader geographic areas and focus on extending this investigation to other clinical settings beyond the medical department, where nursing care complexity and resource needs may differ significantly. For instance, future studies in surgical wards, often characterized by fluctuating care demands, could provide insights into the dynamic relationship between nursing care complexity and resource allocation models.

The health policy implications of these findings are significant. A health system that fails to adequately account for nursing complexity not only risks undermining care quality but may also lead to inefficiencies in resource use. By integrating tools like SIPI into reimbursement and staffing systems, health policies could more accurately reflect the true care needs, improving patient outcomes, optimizing the distribution of resources, and favoring a greater contribution of nursing to health outcomes and care quality [31]. Further research should include broader geographic areas and other clinical settings.

## Figures and Tables

**Table 1:** Patients’ characteristics at admission (N admissions = 6872; N patients = 5129).

Variables	Overall Median [IQR] or N (%)	SIPI < 50 (N = 3950) Median [IQR] or N (%)	SIPI ≥ 50 (N = 2922) Median [IQR] or N (%)
**Age**, years	76 [64, 84]	70.5 [57, 79]	81 [73, 87]
**Biological Sex**			
M	3647 (53.1)	1717 (43.5)	1508 (51.6)
F	3225 (46.9)	2233 (56.5)	1414 (48.4)
**Ward**			
Low intensity	4530 (65.9)	1373 (34.8)	969 (33.2)
Medium intensity	2342 (34.1)	2577 (65.2)	1953 (66.8)
**Access**			
Ordinary	573 (8.3)	496 (12.6)	77 (2.6)
Emergency	6299 (91.7)	3454 (87.4)	2845 (97.4)
**CCI**			
0	1677 (24.4)	1110 (28.1)	568 (19.4)
1	3408 (49.6)	1770 (44.8)	1232 (42.2)
2	1355 (19.7)	871 (22.1)	803 (27.5)
3	380 (5.5)	175 (4.4)	273 (9.3)
≥4	52 (0.7)	24 (0.6)	46 (1.6)
**SIPI**	42.70 [23.40, 68.80]	26.10 [16.20, 37.58]	71.40 [61.70, 81.10]
≥50	3950 (57.5)		
<50	2922 (42.5)		
**DRG rate**, EUR	3325 [2445, 4145]	3285 [2298, 4052]	3536 [2805, 4278]
**Nurse staffing**, minutes per patient per day (Clinical Unit)			
120 (Medicine)	1649 (24.0)	722 (18.3)	927 (31.7)
180 (Nefrology, Oncology, SOAP)	4409 (64.2)	2638 (66.8)	1771 (60.6)
240 (Infectious Diseases)	814 (11.8)	590 (14.9)	224 (7.7)
**Length of stay at discharge**, days	11 [7, 18]	9 [6, 15]	13 [8, 21]
**Death during hospital stay**	469 (6.8)	74 (1.9)	395 (13.5)

Note: CCI = Charlson Comorbidity Index. SIPI = Information System of Nursing Performance: scores ≥ 50 correspond to a high level of complexity of nursing care; scores < 50 correspond to a low level of complexity of nursing care. DRG = Diagnosis-Related Group.

**Table 2:** Patients’ characteristics by nurse staffing in minutes per patient per day.

Variables	Nurse Staffing in Minutes per Patient per Day (Clinical Unit)		
	120 (Medicine) N = 1649 Median [IQR] or N (%)	180 (Nefrology, Oncology, SOAP) N = 4409 Median [IQR] or N (%)	240 (Infectious Diseases) N = 814 Median [IQR] or N (%)
**Age**, years	81 [73, 87]	75 [65, 83]	57 [42, 73]
**Biological Sex**			
M	841 (51.0)	2077 (47.1)	307 (37.7)
F	808 (49.0)	2332 (52.9)	507 (62.3)
**Ward**			
Medium intensity	0 (0.0)	2342 (53.1)	0 (0.0)
Low intensity	1649 (100.0)	2067 (46.9)	814 (100.0)
**Access**			
Ordinary	45 (2.7)	511 (11.6)	17 (2.1)
Emergency	1604 (97.3)	3898 (88.4)	797 (97.9)
**CCI**			
0	377 (22.9)	894 (20.3)	407 (50.0)
1	635 (38.5)	2110 (47.9)	257 (31.6)
2	456 (27.7)	1100 (24.9)	118 (14.5)
3	158 (9.6)	260 (5.9)	30 (3.7)
≥4	23 (1.4)	45 (1.0)	2 (0.2)
**SIPI**	57.00 [27.40, 76.50]	41.30 [24.60, 66.10]	28.30 [21.00, 53.88]
SIPI ≥50	722 (43.8)	2638 (59.8)	590 (72.5)
SIPI <50	927 (56.2)	1771 (40.2)	224 (27.5)
**DRG rate**, EUR	3298 [2734, 4052]	3285 [2437, 4145]	3536 [2445, 8534]
**Length of stay at discharge**, days	14 [10, 21]	9 [6, 15]	10 [6, 19]
**Death during hospital stay**	126 (7.6)	312 (7.1)	31 (3.8)

Note: CCI = Charlson Comorbidity Index. SIPI = Information System of Nursing Performance: scores ≥ 50 correspond to a high level of complexity of nursing care; scores < 50 correspond to a low level of complexity of nursing care. DRG = Diagnosis-Related Group.

**Table 3:** Distribution of DRG rates in EUR by SIPI categories according to deciles.

SIPI Categories (Deciles)	DRG Rates, EUR (Median [IQR])
[0, 13.1]	3063 [2170, 4052]
(13.1, 19.9]	3285 [2184, 4052]
(19.9, 27.1]	3285 [2298, 4052]
(27.1, 34.7]	3285 [2298, 4145]
(34.7, 42.7]	3325 [2445, 4154]
(42.7, 52.6]	3285 [2701, 4052]
(52.6, 63.9]	3311 [2734, 4052]
(63.9, 72.1]	3536 [2792, 4052]
(72.1, 81.1]	3536 [2802, 4278]
(81.1, 98.2]	3791 [3063, 8534]

**Table 4:** Unadjusted and adjusted Cox models with gamma frailty (total number of events: 469) on mortality in 6872 hospital admissions. The hazard ratio of SIPI (>50 vs. ≤50) was estimated separately within two time-intervals: before and after day 10 since admission.

Factors	Unadjusted (N = 6872)		Adjusted (N = 6872)	
	HR (95% CI)	*p* Value	HR (95% CI)	*p* Value
Age, per 10 years	1.483 (1.363; 1.613)	<0.001	1.281 (1.164; 1.410)	<0.001
Gender, Male vs. Female	1.050 (0.875; 1.260)	0.640	1.208 (1.001; 1.458)	0.049
Ward, Medium Intensity vs. Low	1.612 (1.333; 1.949)	<0.001	1.262 (0.992; 1.605)	0.058
Access, Emergency vs. Ordinary	5.037 (2.086; 12.160)	<0.001	3.553 (1.437; 8.786)	0.006
CCI, 1 vs. 0	1.609 (1.238; 2.091)	<0.001	1.411 (1.077; 1.848)	0.012
CCI, ≥2 vs. 0	1.654 (1.252; 2.186)	<0.001	1.396 (1.061; 1.836)	0.017
SIPI > 50, vs. ≤50 at ≤10 days since admission	7.907 (5.450; 11.473)	<0.001	6.222 (4.254; 9.100)	<0.001
SIPI > 50, vs. ≤50 at >10 days since admission	2.868 (2.048; 4.016)	<0.001	2.456 (1.741; 3.464)	<0.001
DRG rates, per EUR 1000	1.007 (0.995; 1.019)	0.280	1.018 (1.004; 1.032)	0.014
Nurse staffing, minutes per patient per day 180 vs. 120	1.347 (1.094; 1.658)	0.005	1.545 (1.197; 1.994)	0.008
Nurse staffing, minutes per patient per day 240 vs. 120	0.627 (0.423; 0.929)	0.020	1.091 (0.727; 1.636)	0.670

Note: CI = Confidence Interval. CCI = Charlson Comorbidity Index. SIPI = Information System of Nursing Performance: scores ≥ 50 correspond to a high level of complexity of nursing care; scores < 50 correspond to a low level of complexity of nursing care. DRG = Diagnosis-Related Group.

## Data Availability

Research data other than those presented in this manuscript can be made available upon request to the corresponding author.

## Supplementary Materials

The following supporting information can be downloaded at https://www.mdpi.com/article/10.3390/healthcare12191988/s1, Figure S1: Scatterplot of the association between SIPI and DRG rates.
