# Supplementary material for: How Are Diagnosis-Related Groups and Staffing Allocation Systems Associated with the Complexity of Nursing Care? An Observational Study
Source: Healthcare (Basel). 2024 Oct 5;12(19):1988. doi: 10.3390/healthcare12191988 (PMC11475643; doi:10.3390/healthcare12191988)
Supplement: Supplementary file 1 [file healthcare-12-01988-s001.zip › healthcare-3150056-SI.pdf]

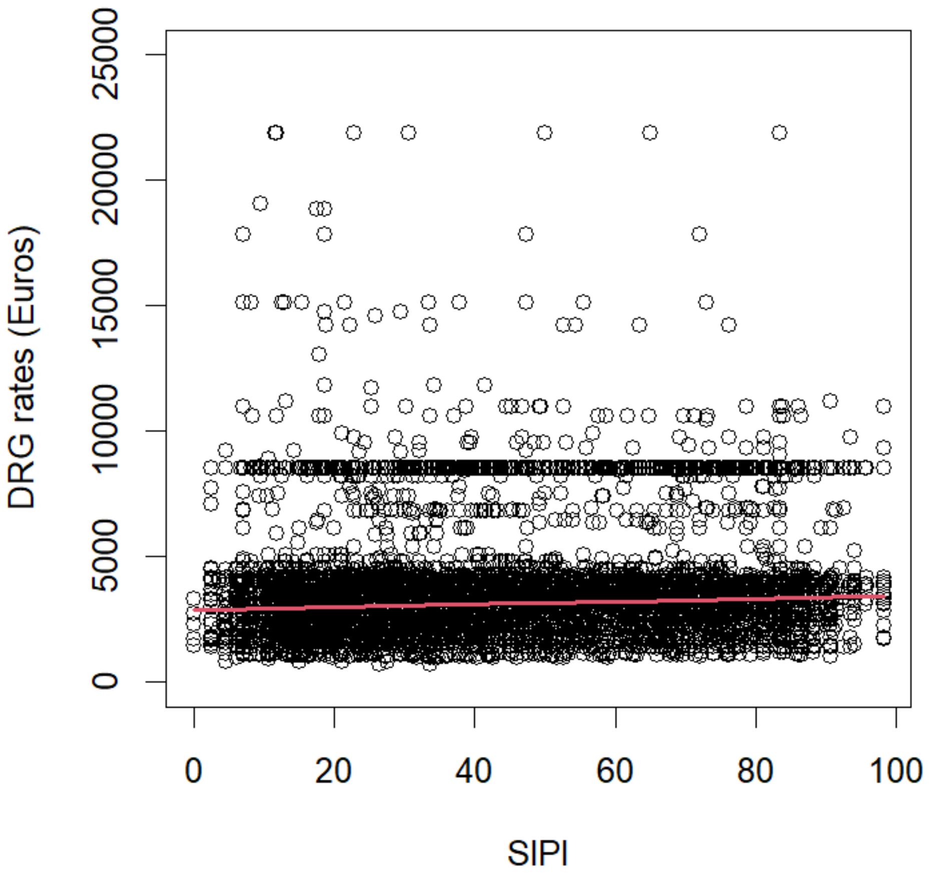

Figure S1. Scatter plot of the association between SIPI and DRG rates. A smoothed tendency line is also shown in red.
